# Artificially Sweetened Food Mediates the Perception of Chronic Pain in Individuals With Neuroticism Traits: A Mendelian Randomization Study

**DOI:** 10.1002/brb3.70476

**Published:** 2025-04-10

**Authors:** Huanghong Zhao, Dongsheng Guan, Zhen Ma, Minghui Yang, Ning Dong, Jian Guo

**Affiliations:** ^1^ Henan Provincial Hospital of Traditional Chinese Medicine Zhengzhou China

**Keywords:** artificially sweetened food, chronic pain, depressed affect, experiencing mood swings, mediation analysis, Mendelian randomization, neuroticism traits, worry

## Abstract

**Background:**

Previous studies have shown that neuroticism and artificially sweetened food all play essential roles in chronic pain to varying degrees. However, it is unclear precisely the causal relationship between neuroticism traits and chronic pain and whether an unhealthy sweetened food is a mediator in this process.

**Methods:**

This study employed rigorous research methods to ensure the validity of the findings. We utilized Mendelian randomization (MR) to examine the causal relationships between neuroticism traits, artificially sweetened food, and chronic pain. The data encompass four neuroticism traits (neuroticism, experiencing mood swings, depressed affect, and worry), consumption levels of nine artificially sweetened foods, and seven types of chronic pain. The primary statistical method employed was inverse variance weighting (IVW). Eventually, we explored whether artificially sweetened food serves as a mediator in the relationship between neuroticism traits and chronic pain.

**Results:**

We found that genetic predisposition to higher neuroticism traits and the consumption of artificial sweeteners is associated with an increased risk of chronic pain across multiple sites. Reverse MR analysis also confirms that chronic pain at multiple sites similarly increases the risk of neuroticism traits. Two‐step MR suggests the mediating effects of five artificial sweeteners on sciatica: low back pain, thoracic pain, low back pain, joint pain, and muscular pain. These findings could inform interventions and treatments for chronic pain.

**Conclusion:**

Neuroticism traits and chronic pain have causal relationships, with artificially sweetened food mediating the pathway from neuroticism traits to chronic pain.

## Graphical abstract

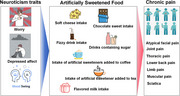

## 1 Introduction

Neuroticism, characterized by emotional instability, increases susceptibility to depression and anxiety, which are closely linked to chronic pain (Meng et al. 2020). Studies show that neuroticism traits predict more severe depression when protective factors like self‐compassion are lacking (Ashina et al. 2017). Additionally, the bidirectional relationship between chronic pain and mental health disorders, such as anxiety and depression, highlights how each can intensify the other. Improvements in mental health can reduce pain perception, suggesting that psychological and physical health are deeply interconnected (Harkins et al. 1989). Populations with chronic pain often experience higher rates of anxiety and depression, indicating that addressing these mental health issues is vital for effective chronic pain management (Chen et al. 2023). Consequently, treatment strategies considering psychological resilience and physical symptoms are essential for better patient outcomes.

Individuals with high levels of neuroticism often exhibit specific dietary preferences related to their psychological traits (Grach et al. 2023). Research has indicated that neuroticism can influence dietary choices, leading to a preference for sweet flavors, potentially as a form of self‐medication for emotional regulation (Low et al. 2017). This preference for sweetened products, including nonnutritive sweeteners, may be linked to internal physiological mechanisms regulating mood and stress response systems (Miller and Branscum 2023). These preferences are likely rooted in neurobiological pathways that are particularly sensitive to stress and emotional dysregulation, often exacerbated in individuals with high neuroticism (Hoerr et al. 2017). Research has indicated that neuroticism traits can influence dietary choices, which may be self‐medication for emotional regulation (Ohara et al. 2019). These preferences are incidental and linked to internal physiological mechanisms regulating mood and stress response systems (Klucken et al. 2019).

Consuming artificially sweetened foods and having a preference for sweets can influence chronic pain via various physiological pathways (Haga et al. 2023). Unlike glucose, sucrose, and fructose, artificially sweetened foods like aspartame and sucralose typically do not induce feelings of fullness but instead stimulate appetite. This “nonphysiological appetite” can significantly tax intestinal metabolism. Studies have shown that unusual alterations in the gut microbiota of individuals with a sweet tooth can influence the body's pain response mechanism (Steinert et al. 2011). One factor is that long‐term consumption of diets high in artificial sweeteners may induce inflammation, which can either exacerbate or trigger existing pain conditions, a critical factor in chronic pain (Basson et al. 2021). Specifically, sweeteners like aspartame have been observed to potentially trigger headaches, a common type of chronic pain, due to their excitotoxic effects on neuronal and vascular function (Hunter et al. 2019). Therefore, given that artificially sweetened foods do not readily induce feelings of fullness and instead stimulate appetite, this not only alters individuals' eating behaviors but also increases the intestinal burden due to excessive caloric and fatty intake, disrupts microbiota balance, and consequently exerts a significant influence on health outcomes, including persistent pain.

Genome‐wide association studies (GWAS) examine millions of genetic variants in an individual's genome, providing insights into complex diseases. Mendelian randomization (MR) is less affected by environmental factors and reverse causality and is a robust genetic epidemiological tool (Birney 2022). In MR studies, genetic variation is used as an instrumental variable (IV) to assess causality between exposures and outcomes, and, unlike typical observational studies, it utilizes pooled estimates of exposures and outcomes from genetic databases from different sources to improve statistical power and thus enhance the assessment of causal effects between exposures and outcomes (Bowden and Holmes 2019).

In this study, we conducted a comprehensive MR analysis of the causal relationship between neuroticism traits and chronic pain, and through reverse causality analysis, we investigated whether genetic predisposition to risk of chronic pain influences neuroticism traits. In addition, we investigated whether artificially sweetened food mediates the pathway from neuroticism traits to chronic pain.

## 2 Methods

### 2.1 Study Design

**FIGURE 1:**
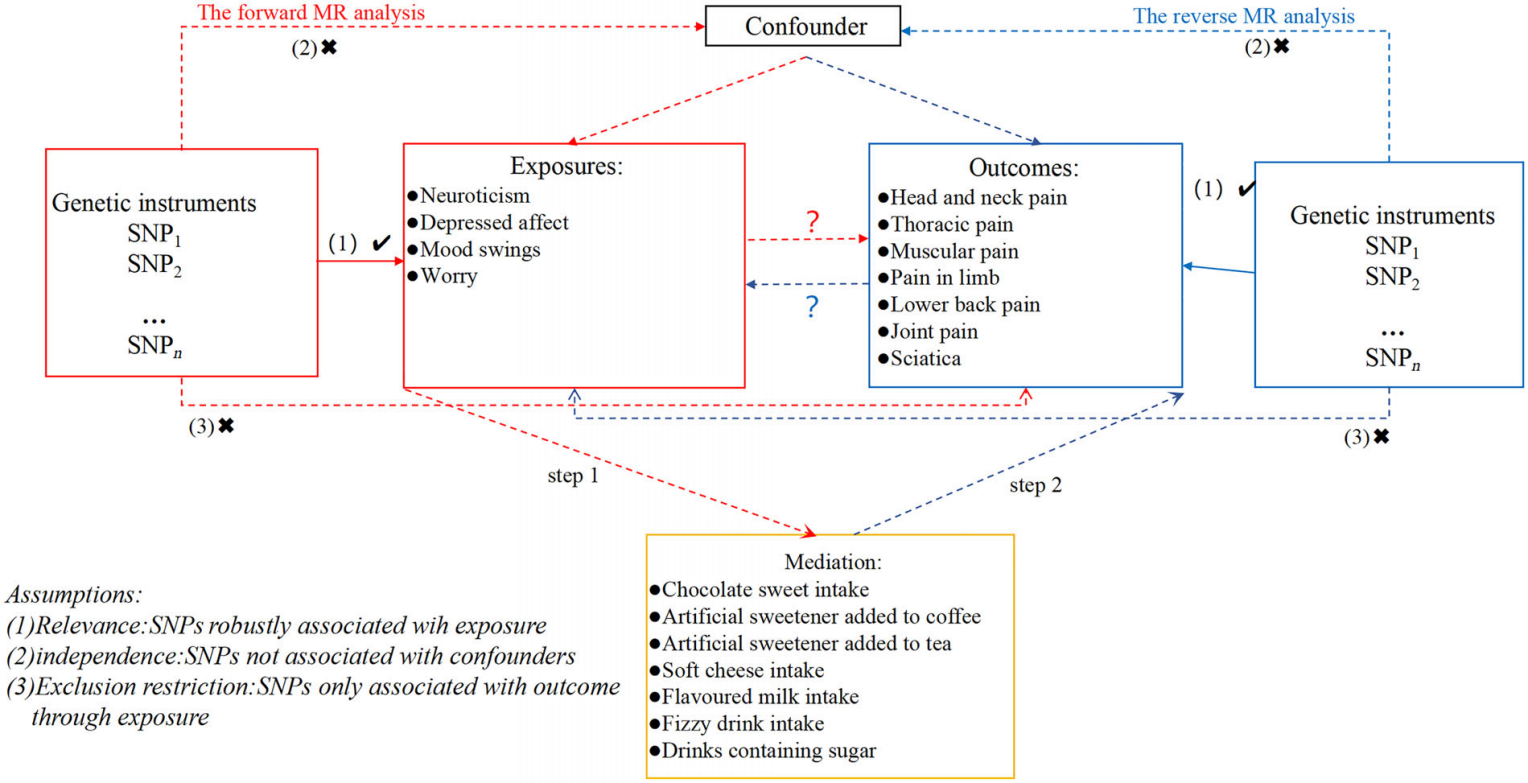
Research and design flowchart for articles.

Our study provided a comprehensive flowchart illustrating the design of the study in detail (Figure 1). First, we performed two‐sample bidirectional resonance analyses based on a recent large‐scale GWAS of individuals of European ancestry, followed by a two‐step magnetic resonance approach to assess potential of relationships between neuroticism traits and chronic pain and assess the potential artificially sweetened food as the mediation in these relationships. This paper is a secondary analysis of publicly available summaries of GWAS data analyses. Ethical approval was obtained for each of the original GWAS studies, and no individual‐level data were used in this investigation, so no further ethical review board consent was required.

### 2.2 Data Source

Genetic data on neuroticism were sourced from the SSGAC consortium in a European population of 933,970 individuals (Okbay et al. 2016). The GWAS study on mood swings involved 373,733 individuals of European ancestry, aiming to elucidate the genetic diversity of neuroticism traits (Nagel et al. 2018). The meta‐analysis of genome‐wide associations for depressive mood and worry involved 449,484 European neurotic individuals (Nagel et al. 2018).

The phenotype data related to artificially sweetened food were obtained from the UK Biobank (https://www.ukbiobank.ac.uk), including sweets intake (*N* = 64,949), chocolate sweet intake (*N* = 64949), soft cheese intake (*N* = 64949), flavored milk intake (*N* = 64,949), fizzy drink intake (*N* = 64,941), drinks containing sugar (*N* = 461,046), intake of artificial sweetener added to cereal (*N* = 64,949), intake of artificial sweetener added to coffee (*N* = 64,949), and intake of artificial sweetener added to tea (*N* = 64,949).

Data for atypical facial pain (1508 cases and 360,538 controls), thoracic pain (5187 cases and 29,4770 controls), limb pain (189,683 cases and 221,680 controls), joint pain (30,614 cases and 222,498 controls), lower back pain (46,707 cases and 365,474 controls), and sciatica (46,707 cases and 365,474 controls) were all obtained from the 8th version of the FinnGen consortium (https://r8.risteys.fnngen.fi/). Detailed information on participants for each GWAS database is provided in Table 1.

**TABLE 1:** Detailed information on included traits in the present study.

Traits	Ancestry	Consortium	Sample size(cases/controls)	Data source
Neuroticism traits				
Neuroticism	European	SSGAC	170,911	ieu‐a‐1007
Experiencing mood swings	European	NA	373,733	ebi‐a‐GCST006944
Depressed affect	European	NA	357,957	ebi‐a‐GCST006475
Worry	European	NA	348,219	ebi‐a‐GCST006478
Mediations				
Sweets intake	European	MRC‐IEU	649,49	ukb‐b‐10217
Chocolate sweet intake	European	MRC‐IEU	64,949	ukb‐b‐9835
Soft cheese intake	European	MRC‐IEU	64,949	ukb‐b‐8007
Flavored milk intake	European	MRC‐IEU	64,941	ukb‐b‐12763
Fizzy drink intake	European	MRC‐IEU	64,949	ukb‐b‐2832
Drinks containing sugar	European	MRC‐IEU	461,046	ukb‐b‐5495
Intake of artificial sweeteners added to cereal	European	MRC‐IEU	64,949	ukb‐b‐3143
Intake of artificial sweeteners added to coffee	European	MRC‐IEU	64,949	ukb‐b‐1338
Intake of artificial sweetener added to tea	European	MRC‐IEU	64,949	ukb‐b‐5867
Chronic pain				
Atypical facial pain	European	FinnGen	362,046(1508/360,538)	https://r8.finngen.fi/
Thoracic pain	European	FinnGen	299,957(5187/294,770)	https://r8.finngen.fi/
Limb pain	European	FinnGen	411,363(189683/221,680)	https://r8.finngen.fi/
Muscular pain	European	FinnGen	412,181(10,243/401,938)	https://r8.finngen.fi/
Joint pain	European	FinnGen	253,112(30,614/222,498)	https://r8.finngen.fi/
Lower back pain	European	FinnGen	412,181(46,707/365,474)	https://r8.finngen.fi/
Sciatica	European	FinnGen	412,181(46,707/365,474)	https://r8.finngen.fi/

### 2.3 IVs Selection

Our research found that single nucleotide polymorphisms (SNPs) are significantly associated with neuroticism, with a significance level below 5 × 10^−6^. We set a strict SNP selection threshold of 5 × 10^−6^ to utilize existing genetic tools fully. We excluded any SNPs exhibiting linkage disequilibrium (LD) with a cut‐off value of *r* ^2^ < 0.001 and isolated SNPs exceeding 10,000 kb from our analysis. Therefore, we removed SNPs that met these criteria to ensure the robustness of our research results (Widding‐Havneraas and Zachrisson 2022).

### 2.4 MR Analysis

#### 2.4.1 Primary Analysis

As indicated in Figure 1, we employ two‐sample MR analyses to evaluate the causal impact of neuroticism and artificially sweetened food on chronic pain. The Wald ratios test was applied to the IVW approach as a primary statistical method for variables having a single IV. The 95% confidence intervals (CIs) and the odds ratios (ORs) of the MR outcomes were given. When the IVW method's *p* value was lower than 0.05, and the directions for both IVW and MR‐Egger matched up, significance was obtained (Burgess et al. 2017).

#### 2.4.2 Mediation Analysis

The neuroticism and the substantial impact artificially sweetened food on chronic pain were included in the mediation analysis as part of the two‐sample analysis. To determine if artificially sweetened food was a mediator in the pathway from neuroticism to this disease, we used multiple MR analyses on the idea that neuroticism had a causal effect on artificially sweetened food (Steps 1 and 2 in Figure 1).

#### 2.4.3 Bidirectional Causality Analysis

We treated chronic pain as a form of “exposure” and neuroticism or artificially sweetened food caused by the disorders as the overall “outcome” for evaluating the bidirectional causation impacts between neuroticism, artificially sweetened food, and chronic pain. As IVs, we chose the SNPs that had a strong relationship with chronic pain *(p *< 5 × 10^−6^).

#### 2.4.4 Sensitivity Analysis

In each phase of our study, we utilized R (version 4.3.2) as our statistical analysis software. The MR analyses were performed with the TwoSampleMR package in R. We conducted the Cochran's *Q* test to assess heterogeneity among SNPs (Cohen et al. 2015). Scatter plots were generated to display the relationships between SNPs and exposure, highlighting the outcomes of the MR study.

In the leave‐one‐out analysis (LEO‐A), each SNP was systematically excluded one at a time to test its influence on the outcome. Subsequently, the Inverse Variance Weighted (IVW) method was applied to the remaining SNPs to evaluate their potential impact on our predictions (Gurung et al. 2020). We also explored the possibility of horizontal pleiotropy using MR‐PRESSO and MR‐Egger regression techniques. MR‐PRESSO identified significant outliers, and their removal was crucial for adjusting the effects related to horizontal pleiotropy (Plana et al. 2021).

## 3 Results

### 3.1 Genetic Instruments

The initial group of SNPs we discovered, at a significance level of *p* < 5 × 10^−6^, was associated with neuroticism (68 SNPs), experiencing mood swings (72 SNPs), depressed affect (190 SNPs), and worry (195 SNPs), respectively. These 525 SNPs were subsequently selected as IVs (see Tables S1–S4). At the same significance threshold (*p* < 5 × 10^−6^), we identified 497 SNPs linked to artificially sweetened food (see Table S5). All selected SNPs exhibited an *F*‐statistic value exceeding 10, indicating a minimal risk of weak instrument bias.

#### 3.1.1 The Causal Effects of Neuroticism on Chronic Pain

**FIGURE 2:**
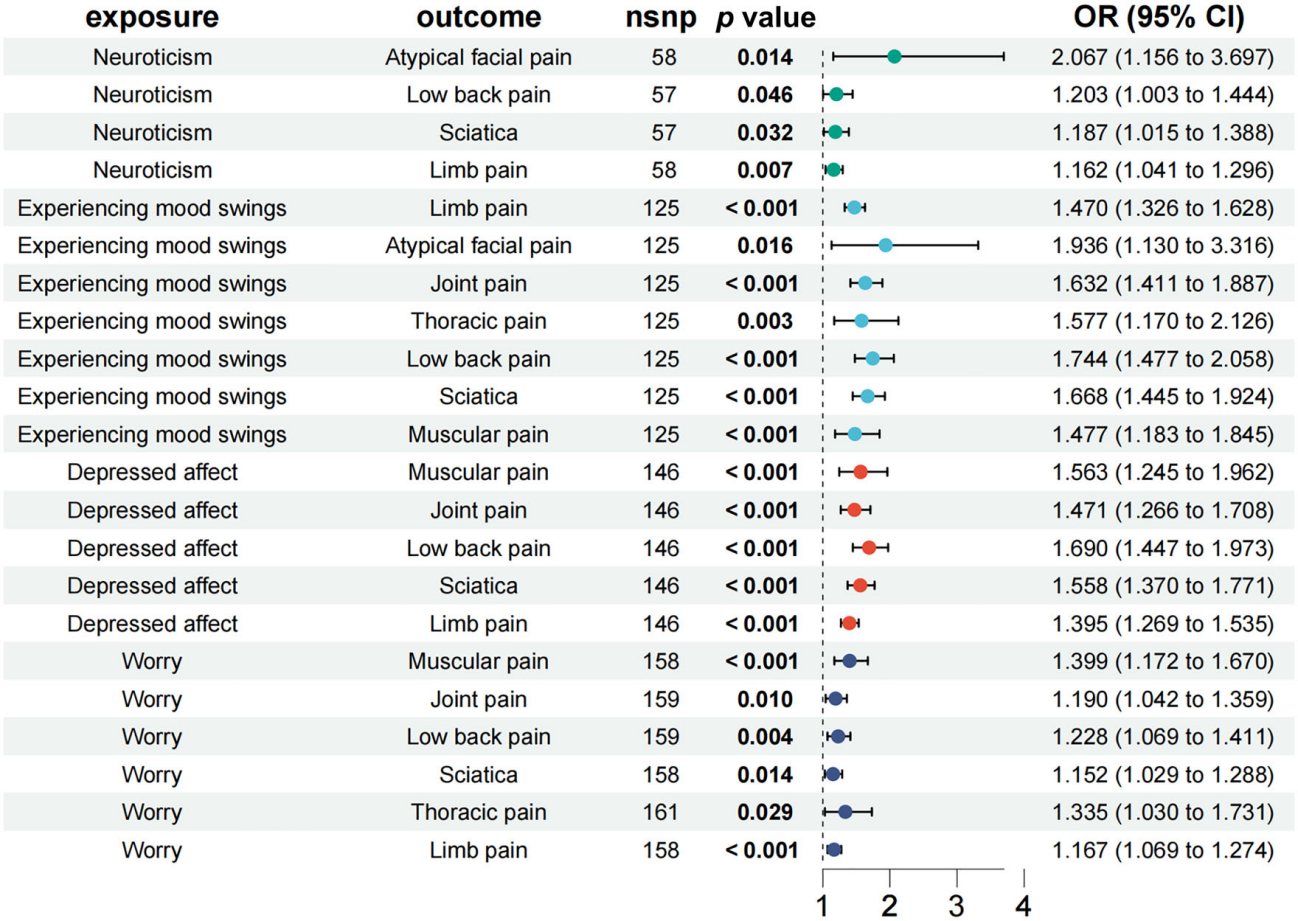
The causal effects of neuroticism on chronic pain.

As illustrated in Figure 2, MR analysis reveals that atypical facial pain was associated with a significant risk of neuroticism (OR = 2.067, 95% CI = 1.15–3.697, *p = *0.014). Limb pain (OR = 1.470, 95% CI = 1.326–1.628, *p* < 0.001), atypical facial pain (OR = 1.936, 95% CI = 1.130–3.316, *p = *0.016), joint pain (OR = 1.632, 95% CI = 1.411–1.887, *p* < 0.001), thoracic pain (OR = 1.577, 95% CI = 1.170–2.216, *p = *0.003), low back pain (OR = 1.744, 95% CI = 1.477–2.058, *p* < 0.001), sciatica (OR = 1.668, 95% CI = 1.445–1.924, *p* < 0.001), and muscular pain (OR = 1.477, 95% CI = 1.183–1.845, *p* < 0.001) were identified as all increasing the incidence of experiencing mood swings. Muscular pain (OR = 1.563, 95% CI = 1.245–1.962, *p* < 0.001), joint pain (OR = 1.471, 95% CI = 1.266–1.708, *p* < 0.001), low back pain (OR = 1.690, 95% CI = 1.447–1.973, *p* < 0.001), sciatica (OR = 1.558, 95% CI = 1.370–1.771, *p* < 0.001) limb pain (OR = 1.395, 95% CI = 1.269–1.535, *p* < 0.001) were identified as all increasing the incidence of depressed affect. Muscular pain was associated with a significant worry risk (OR = 1.399, 95% CI = 1172–1.670, *p* < 0.001).

#### 3.1.2 The Causal Effects of Chronic Pain on Neuroticism

**FIGURE 3:**
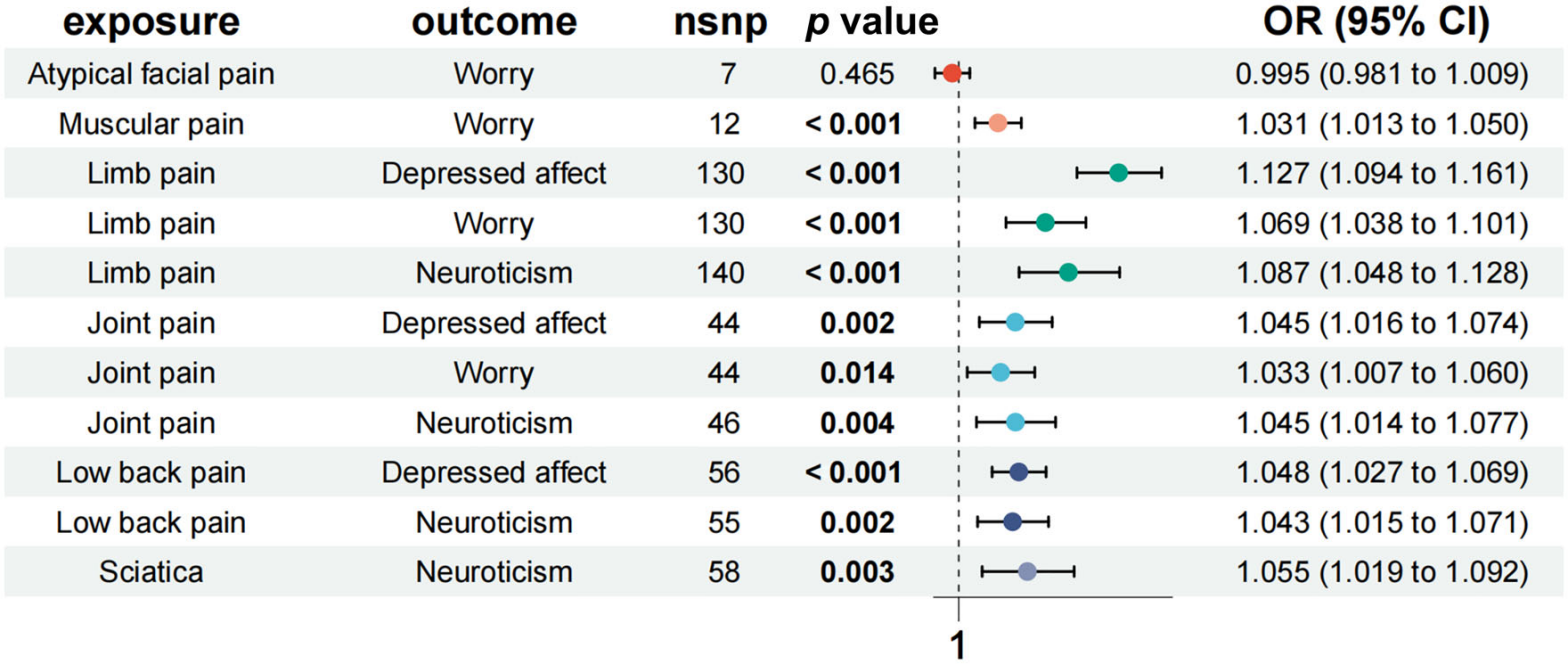
The causal effects of chronic pain on neuroticism.

As illustrated in Figure 3, MR analysis reveals that depressed affect was associated with limb pain (OR = 1.127, 95% CI = 1.094–1.161, *p < *0.001), joint pain (OR = 1.045, 95% CI = 1.016–1.074, *p = *0.002), and low back pain (OR = 1.048, 95% CI = 1.027–1.069, *p < *0.001). The incidence of worry was associated with muscular pain (OR = 1.031, 95% CI = 1.013–1.050, *p < *0.001), limb pain (OR = 1.069, 95% CI = 1.038–1.101, *p < *0.001) joint pain (OR = 1.033, 95% CI = 1.007–1.060, *p = *0.014). The occurrence of neuroticism was associated with limb pain (OR = 1.087, 95% CI = 1.048–1.128, *p < *0.001), joint pain (OR = 1.045, 95% CI = 1.014–1.077, *p = *0.004), low back pain (OR = 1.043, 95% CI = 1.015–1.071, *p = *0.002) sciatica (OR = 1.055, 95% CI = 1.019–1.092, *p = *0.003).

#### 3.1.3 The Causal Effects of Neuroticism on Artificially Sweetened Food

**FIGURE 4:**
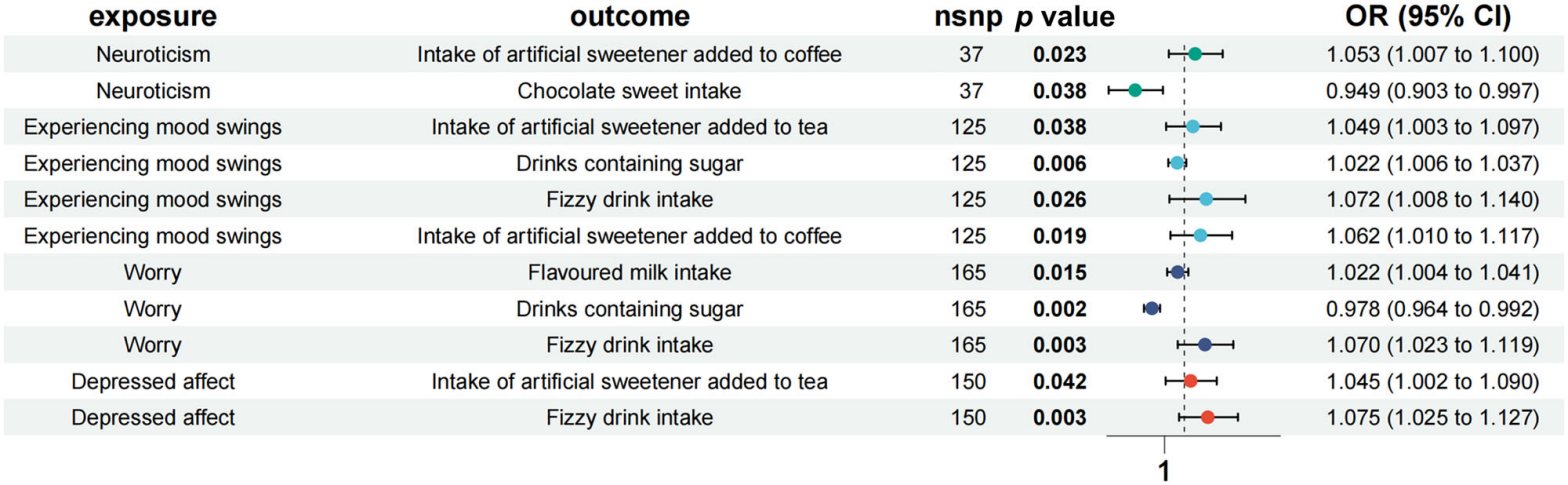
The causal effects of neuroticism on artificially sweetened food.

As illustrated in Figure 4, MR analysis reveals that neuroticism was associated with the intake of artificial sweeteners added–coffee (OR = 1.053, 95% CI = 1.007–1.100, *p = *0.023) and the intake of chocolate sweets (OR = 0.949, 95% CI = 0.903–0.997, *p = *0.038). Experiencing mood swings was associated with the intake of artificial sweetener added–tea (OR = 1.049, 95% CI = 1.003–1.097, *p = *0.038), drinks containing sugar (OR = 1.022, 95% CI = 1.006–1.037, *p = *0.006), fizzy drink intake (OR = 1.072, 95% CI = 1.008–1.140, *p = *0.026), and intake of artificial sweetener added–coffee (OR = 1.062, 95% CI = 1.010–1.117, *p = *0.019). Worry was associated with flavored milk intake (OR = 1.022, 95% CI = 1.004–1.041, *p = *0.015), drinks containing sugar (OR = 0.978, 95% CI = 0.964–0.992, *p = *0.002), and fizzy drink intake (OR = 1.045, 95% CI = 1.002–1.090, *p = *0.003). Depressed affect was associated with intake of artificial sweetener added–tea (OR = 1.045, 95% CI = 1.002–1.090, *p = *0.042) and fizzy drink intake (OR = 1.075, 95% CI = 1.025–1.127, *p = *0.003).

#### 3.1.4 The Causal Effects of Artificially Sweetened Food on Chronic Pain

**FIGURE 5:**
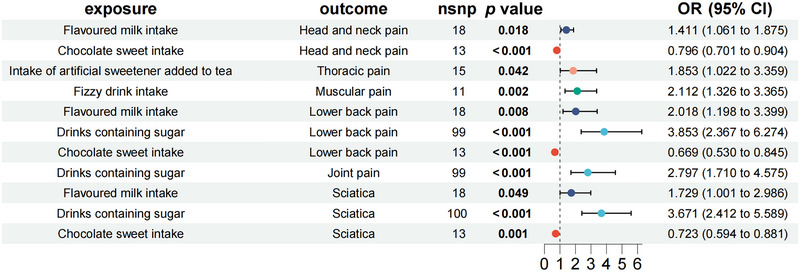
The causal effects of artificially sweetened food on chronic pain.

As illustrated in Figure 5, MR analysis reveals that flavored milk intake was associated with head and neck pain (OR = 1.411, 95% CI = 1.061–1.875, *p = *0.018), lower back pain (OR = 2.018, 95% CI = 1.198–3.399, *p = *0.008), and sciatica (OR = 1.729, 95% CI = 1.001–2.986, *p = *0.049). Chocolate sweet intake was associated with head and neck pain (OR = 0.796, 95% CI = 0.701–0.904, *p* < 0.001) and sciatica (OR = 0.723, 95% CI = 0.594–0.881, *p = *0.001). Intake of artificial sweeteners added–tea was associated with thoracic pain (OR = 1.853, 95% CI = 1.022–3.359, *p = *0.042). Fizzy drink intake was associated with head and neck pain (OR = 2.212, 95% CI = 1.326–3.365, *p = *0.002). Drinks containing sugar were associated with lower back pain (OR = 3.853, 95% CI = 2.367–6.274, *p* < 0.001), joint pain (OR = 2.797, 95% CI = 1.710–4.575, *p* < 0.001), and sciatica (OR = 3.671, 95% CI = 2.412–5.589, *p* < 0.001).

### 3.2 Sensitivity Analyses

The MR‐Egger regression intercept method results showed that genetic pleiotropy influenced them. Additionally, the MR‐PRESSO test established no horizontal pleiotropy in our MR analyses. Cochran's Q test showed no significant heterogeneity among the studies (*p *> 0.05; see Table S6 for details).

The robustness of the MR results was further bolstered by a “leave‐one‐out” sensitivity analysis, a rigorous method that showed consistent reliability (total CIs for SNPs did not include the null hypothesis line). Scatter plots vividly described the cumulative effect of neuroticism on the risk of chronic pain traits. In contrast, the forest plot clearly illustrated a direct causal relationship between neuroticism and the occurrence of chronic pain traits (see Figure 2 for details).

### 3.3 Mediation Analysis

Utilizing a two‐step MR method for mediation analysis, we present in Table 2 the mediation outcomes and proportions attributable to specific factors. Our research findings disclose that chocolate consumption differentially mediates sciatica (9.9%) and lower back pain (11.3%) in neurotic individuals. The intake of fizzy drinks is associated with emotional fluctuations and muscle pain (13.8%), and muscle pain induced by depressive emotions is also linked to fizzy drink consumption (12.0%). Additionally, our survey suggests that consuming flavored milk significantly mediates the relationship between worry and pain (lower back pain, sciatica, muscle pain) (4.9%–8.6%). Similarly, drinks containing sugar to varying degrees mediate the relationship between mood swings and pain (lower back pain, joint pain, sciatica) (1.7%–2.0%).

**TABLE 2:** Multivariable Mendelian randomization analyses of the causal effects between neuroticism traits, artificially sweetened food, and chronic pain.

			Mediation effect			
Exposure	Outcome	Mediator	Direct effect (β1* ± SE)	Indirect effect (ɑ* ± SE)	Indirect effect (β2* ± SE)	Proportion mediated (ɑ × β2*/β1)
Neuroticism	Sciatica	Chocolate sweet intake	0.171 ± 0.080	‘‐0.052 ± 0.025	’‐0.324 ± 0.101	0.099
Neuroticism	Low back pain	Chocolate sweet intake	0.185 ± 0.193	’‐0.052 ± 0.025	‘‐0.402 ± 0.119	0.113
Experiencing mood swings	Thoracic pain	Intake of artificial sweetener added to tea	0.456 ± 0.152	0.023 ± 0.038	0.617 ± 0.304	0.034
Experiencing mood swings	Low back pain	Drinks containing sugar	0.556 ± 0.085	0.008 ± 0.006	1.349 ± 0.249	0.019
Experiencing mood swings	Joint pain	Drinks containing sugar	0.49 ± 0.075	0.008 ± 0.006	1.029 ± 0.251	0.017
Experiencing mood swings	Sciatica	Drinks containing sugar	0.511 ± 0.073	0.008 ± 0.006	1.301 ± 0.214	0.020
Experiencing mood swings	Muscular pain	Fizzy drink intake	0.39 ± 0.113	0.069 ± 0.031	0.748 ± 0.238	0.138
Depressed affect	Muscular pain	Fizzy drink intake	0.447 ± 0.116	0.072 ± 0.024	0.748 ± 0.238	0.120
Worry	Low back pain	Flavored milk intake	0.206 ± 0.071	0.022 ± 0.009	0.702 ± 0.266	0.075
Worry	Sciatica	Flavored milk intake	0.141 ± 0.057	0.022 ± 0.009	0.548 ± 0.279	0.086
Worry	Muscular pain	Flavored milk intake	0.336 ± 0.09	0.022 ± 0.009	0.748 ± 0.238	0.049

Beta (β), standard errors (SE), and *p*‐values were obtained from multivariable Mendelian randomization analysis. β1* and β2* represent the controlled direct effects of each pair of neuroticism and artificially sweetened food on chronic pain after adjusting for each other. α is the causal effect of exposure on the mediator; the indirect effect (α × β2*) is the effect of exposure on chronic pain via the corresponding mediator; β1 is the total effect of exposure on chronic pain; the proportion mediated is calculated as the “indirect effect/total effect.”

## 4 Discussion

In this study, we performed a comprehensive MR investigation to unravel the relationships between neuroticism and chronic pain, utilizing large‐scale summary‐level statistics from GWAS. We found that genetic susceptibility to neuroticism, experiencing mood swings, depressed affect, and worry was associated with an increased risk of chronic pain, including atypical facial pain, thoracic pain, limb pain, muscular pain, joint pain, low back pain, and sciatica. In the reverse directional MR, potential causal associations were also identified between specific pain (but not including thoracic pain) and these emotional disorders.

The phenotype of chronic pain in specific body areas may involve complex and multifaceted mechanisms, encompassing factors such as genetic predisposition, lifestyle choices, and other unmeasurable variables (Niederberger 2020). Interindividual differences are among the most crucial factors (Mak and Schneider 2020). Due to the unpredictability and unquantifiability of pain triggers, as well as the need for more objective evidence in clinical settings regarding individual differences leading to chronic pain, there still needs to be a gap in understanding (Edwards 2005). Little research has been conducted to elucidate whether neuroticism‐related characteristics serve as precursors to chronic pain episodes (Mogil 2021). Therefore, our study aims to contribute objective clinical evidence by exploring whether neuroticism traits can serve as prodromes for chronic pain episodes.

Neuroticism is often characterized by emotional instability, susceptibility to anxiety, the tendency to worry, and a propensity to experience negative emotions, and these psychological factors are closely associated with chronic pain (Zis et al. 2017). Numerous studies have found that individuals high in neuroticism are more prone to experiencing pain, and their cognitive and coping mechanisms for pain may also differ (Hall et al. 2018). Additionally, worry and anxiety have been identified as standard psychological features among chronic pain patients, which may exacerbate the perception of pain and impact patients' coping strategies and treatment outcomes (Snyder and Handrup 2018).

Our reverse MR analysis aims to investigate the impact of chronic pain on neuroticism traits. Chronic pain in specific body areas can predict future neuroticism susceptibility. These findings underscore that persistent bodily pain is not merely discomfort but can also affect deeper emotional and psychological states through bodily mechanisms. Various factors can mediate emotional, psychological, and even pain‐induced personality changes (Tang et al. 2021). Physiological factors may play a significant role. For instance, prolonged pain can lead to physical fatigue and decreased sleep quality, impacting neurotransmitter levels in the brain and consequently affecting emotional and affective states (Magnuson et al. 2016). Social support and environmental factors may also play a role. Individuals lacking support systems and social connections may be more prone to negative emotions (Markus et al. 2021). Individual coping strategies and psychological resilience are also crucial factors. Some individuals may cope with pain through positive psychological mechanisms, such as seeking support and adopting positive lifestyle choices, thus alleviating the impact of negative emotions (Rother et al. 2018). In contrast, others may be more susceptible to negative emotional states.

In our intermediary mediation analysis, we further quantified the role of artificial sweetener foods as mediators in the relationship between neuroticism traits and chronic pain. Nine sweetener foods were selected for this study. Our findings indicate that five sweetener foods partially mediate potential pains (such as sciatica, low back pain, thoracic pain, low back pain, joint pain, and muscular pain). Existing epidemiological studies have also validated that sweetener intake increases the risk of pain. For instance, in a study tracking the dietary intake of over twenty thousand participants for nine years, those consuming more than one serving of artificial sweeteners daily had over twice the risk of developing chronic headaches compared to those who did not use artificial sweeteners (Patel et al. 2006). This association suggests that artificial sweeteners may adversely affect the nervous system, thus increasing the risk of chronic pain. Another study also showed that a calorie‐free sweetener diet (c‐NCS) was prone to triggering functional gastrointestinal disease symptoms (FGDs), such as epigastric burning sensation or retrosternal pain, compared to a calorie‐free sweetener‐free diet (NCS‐f) (Mendoza‐Martínez et al. 2022). In clinical practice, some doctors have begun to advocate for reducing artificial sweetener intake, such as discontinuing beverages containing aspartame, reducing cheese consumption, and limiting the intake of flavored yogurts as part of treating or preventing chronic pain (Newman and Lipton 2001). Our research findings further strengthen the evidence that genetically predicted sweetener diets are positively correlated with the risk of chronic pain and neurotic traits.

The preliminary findings highlight a bidirectional causal relationship between neuroticism traits and chronic pain, suggesting significant implications for both public health and clinical practice. This achievement provides a theoretical underpinning for interventions aimed at preventing or treating these conditions: Given the observed link between neuroticism traits and chronic pain, interventions targeting neuroticism traits could serve as a means to prevent or manage pain. Early screening and treatment methods for chronic pain are crucial in preventing syndromes in the elderly associated with emotional and personality changes such as neuroticism (Sylvetsky and Rother 2018). Promoting a healthy lifestyle and reducing the intake of artificial sweetener foods hold significant value in reducing the high co‐occurrence of pain and neuroticism traits (Gómez‐Fernández et al. 2021). The primary strength of our current study lies in the meticulous utilization of a bidirectional MR design across two samples, employing three MR methods effectively to mitigate bias, confounding, and reverse causation. Thorough scrutiny of MR assumptions was not just conducted; it was exhaustive and bolstered by large‐sample GWAS data sources, further enhancing study credibility. Additionally, bias was not just controlled; it was meticulously managed by restricting participants to individuals of European ancestry. Moreover, MR‐Egger and MR‐PRESSO analyses revealed no evidence of pleiotropic effects, further solidifying the validity of our findings. Furthermore, our exploration through two‐step MR analysis deepens mechanistic understanding and provides evidence support for preventive strategies.

Despite its pioneering nature, this study is subject to certain limitations that warrant acknowledgment. Our investigation, confined solely to the European population, limits the generalizability of our findings to other demographic cohorts. Although we tried to avoid sample overlap by utilizing different public databases from Finland, UKB, and IEUopen GWAS, the inability to access detailed participant information may impact the robustness of our MR results due to potential sample overlap from vast databases. While correction for multiple comparisons is a standard approach in MR to mitigate Type I error risk, it was not applied in this analysis due to workload constraints, data limitations, and adherence to conventional mediation analysis protocols. Future studies should incorporate robust statistical methods to control the elevated risk of false‐positive findings, thereby enhancing reliability and accuracy. Furthermore, while our focus is on artificial sweetener foods as mediators in the relationship between chronic pain and neuroticism traits, other potential confounding factors may affect our analysis, possibly due to unmeasured or confounded variables. This study mainly explores the potential impact of sweeteners as additives on chronic pain. However, further research is needed to determine whether other additives, such as emulsifiers, colorants, and preservatives, also have similar effects. Exploring this issue provides a new direction for future research, namely, an in‐depth analysis of how different additives may affect the mechanisms of chronic pain. Therefore, further research with more diverse populations and comprehensive datasets must be undertaken to validate and extend our findings.

## 5 Conclusion

In this study, we comprehensively explored whether the neuroticism traits and artificial sweetener foods significantly impact the development of chronic pain. Initially, we clarified the positive causal relationships between neuroticism traits, artificial sweetener foods, and chronic pain and conducted sensitivity analyses to support our findings. Subsequently, we delved into the possibility of a reverse causal effect between the two. We identified five vital artificial sweetener foods that intricately regulate the progression of five types of pain. The findings highlight a causal relationship between neuroticism traits and chronic pain, with artificial sweetener foods mediating this relationship.

## Author Contributions

**Huanghong Zhao**: conceptualization. **Dongsheng Guan**: methodology. **Zhen Ma**: data curation. **Minghui Yang**: investigation. **Ning Dong**: validation. **Jian Guo**: formal analysis.

## Conflicts of Interest

The authors declare no conflict of interest.

### Peer Review

The peer review history for this article is available at https://publons.com/publon/10.1002/brb3.70476

## Ethics Statement

Ethics approval and consent to participate The present study is a secondary analysis of publicly available data. Ethical approval was granted for each of the original GWAS studies. In addition, no individual‐level data were used in this study. Therefore, no new ethical review board approval was required.

## Supporting information



Supporting Information

## Data Availability

All data used in the present study were obtained from GWAS summary statistics that were publicly released by genetic consortia.
